# Examination of Lactic Acid Bacteria to Secretion of Bacteriocins

**DOI:** 10.5195/cajgh.2013.106

**Published:** 2014-01-24

**Authors:** Maira Urazova, Asel Moldagulova, Sandugash Anuarbekova, Altynay Tuyakova, Gulyaim Abitaeva, Elvira Nagyzbekkyzy, Eleonora Bekenova, Serik Shaikhin, Kairtai Almagambetov

**Affiliations:** Republican Collection of Microorganisms, Astana, Kazakhstan

**Keywords:** lactic acid bacteria, antibacterial activity, bioassay, food products, kazy

## Abstract

**Introduction:**

Bacteriocins produced by lactic acid bacteria (LAB) have the potential to cover a very broad field of applications, including the food industry and the medical sector. In the food industry, bacteriocinogenic LAB strains can be used as starter cultures, co-cultures, and bioprotective cultures, which would be used to improve food quality and safety. In the medical sector, bacteriocins of probiotic LAB might play a role in interactions, which take place in human gastrointestinal tract, and contribute to gut health. The aim of this study was the examine the effect of LAB antimicrobial activity.

**Methods:**

LAB were isolated from different commercial and home made products, such as kazy and sour cream. To screen for bacteriocin producing LAB, we used an agar diffusion bioassay, described in a previous study by Dr. Yang, with three modifications in cell-free supernatant (CFS). First we had a clear supernatant, second we adjusted the CFS to pH 6.0 to eliminate acids antimicrobial effects, and third the CFS pH 6.0 was treated with catalase to exclude the action of H_2_O_2_ and confirm action of bacteriocin-like substances. Pathogenic *S.marcescens, E. coli, S.aureus* cultures were used as indicators.

**Results:**

Screening of 95 strains of LAB through deferred antagonism to six indicator cultures showed that all of the selected strains had a high value of antibacterial activity. However, CFS of only 50 strains retained their antimicrobial activity, and 10 of them lost this activity in the second modification of CFS with pH 6.0 to test culture *S.marcescens,* which confirmed the acidic nature of antimicrobial activity of CFS. *Lb.rhamnosus* (P-1), *Lb.fermentum* (N-6), and *Lc.lactis* (7M) lost antibacterial activity in the presence of the catalase. All modifications of CFS of three strains: *Lb.pentosus* (16al), *Lb.pentosus* (P-2), and *Pediococcusacidilactici* (8) retained inhibitory activity to *E.coli* and *S. aureus*. Supernatants of only *Lactococcusgarvieae* (10a) and *Pediococcusacidilactici* (25) extracted from homemade meat food kazy (Karaganda) and sour cream (Astana), respectively retained antibacterial activity to all three indicator cultures.

**Conclusion:**

The antibacterial activity (pH 6.0, added catalase) of *Lactococcusgarvieae* (10a) and *Pediococcusacidilactici* (25) to *S. marcescens*, *E. coli,* and *S.aureus* indicates these strains as promising strains for further use in the preparation of bacteriocins.

